# Realizing ecosystem-safe hydropower from dams

**DOI:** 10.1186/s40807-020-00060-9

**Published:** 2020-06-01

**Authors:** Shahryar Khalique Ahmad, Faisal Hossain

**Affiliations:** Dept. of Civil and Environmental Engineering, Univ. of Washington, More Hall 201, Seattle, WA 98195 USA

**Keywords:** Hydropower, Ecosystem-safe, Temperature change, Remote sensing, Optimization, Regression

## Abstract

For clean hydropower generation while sustaining ecosystems, minimizing harmful impacts and balancing multiple water needs is an integral component. One particularly harmful effect not managed explicitly by hydropower operations is thermal destabilization of downstream waters. To demonstrate that the thermal destabilization by hydropower dams can be managed while maximizing energy production, we modelled thermal change in downstream waters as a function of decision variables for hydropower operation (reservoir level, powered/spillway release, storage), forecast reservoir inflow and air temperature for a dam site with in situ thermal measurements. For data-limited regions, remote sensing-based temperature estimation algorithm was established using thermal infrared band of Landsat ETM+ over multiple dams. The model for water temperature change was used to impose additional constraints of tolerable downstream cooling or warming (1–6 °C of change) on multi-objective optimization to maximize hydropower. A reservoir release policy adaptive to thermally optimum levels for aquatic species was derived. The novel concept was implemented for Detroit dam in Oregon (USA). Resulting benefits to hydropower generation strongly correlated with allowable flexibility in temperature constraints. Wet years were able to satisfy stringent temperature constraints and produce substantial hydropower benefits, while dry years, in contrast, were challenging to adhere to the upstream thermal regime.

## Introduction

The need to satisfy energy demand of a growing planet while simultaneously meeting sustainability standards with clean energy generation has resulted in a growing hydropower infrastructure, especially in the developing regions (Moran et al. 2018). The design and management of such infrastructure has traditionally focused on flood control, hydropower, water supply, and irrigation (Carron and Rajaram 2001). Hydropower, once perceived as clean and renewable, has now become a contributor of negative ecological impacts to the reservoir and aquatic and riparian ecosystem (Abbasi and Abbasi 2000). Hereafter ‘reservoir’ and ‘dam’ are used interchangeably to imply the reservoir-dam system.

Coldwater fishes such as salmon and trout are sensitive to changes in water temperature. Extreme temperature deviations can be lethal to their population (Handcock et al. 2012). Warm water tends to hold less dissolved oxygen which is critical to the health of aquatic habitat (Li et al. 2014). Such adverse thermal impacts of hydropower dam operation demand a reevaluation of dams’ operational objectives from an ecosystem standpoint (U.S. Department of the Interior 1995; McCartney 2009). In the past, recommendations have usually specified minimum flow release from reservoirs for habitat maintenance, water quality, and temperature control (Carron and Rajaram 2001; Chen and Olden 2017). However, little or no recommendation exists in the form of operational strategy to minimize the negative ecosystem impacts from a thermal standpoint. Thus, one of the formidable challenges that exist today and will only intensify in the future with changing climate and increasing hydropower dam construction (Moran et al. 2018; Zarfl et al. 2015) is the alteration of river’s natural thermal regime by the hydropower operations (Olden and Naiman 2010).

### Thermal pollution from hydropower operations

The natural temperature of regulated rivers, apart from responding to changes in hydrologic and hydraulic conditions, is largely impacted by the operations of regulating reservoirs in the upstream (Gu et al. 1999). During the seasons of maximum heat exchange between reservoir surface and atmosphere, the surface warms rapidly lowering its density. The lower density surface rests on top of water column that becomes colder and denser with depth. This inhibits the vertical mixing of reservoir and causes seasonal thermal stratification with low diffusion rates between the top and bottom reservoir layers, also termed as epilimnion and hypolimnion, respectively (Niemeyer et al. 2018; Xie et al. 2017). The surface warming is also enhanced by the large reservoir surface area and resulting longer residence time of the rivers (Vörösmarty et al. 1997). During hydropower operations, penstocks, usually located at the bottom layers (hypolimnion), tend to release cold water and lower the downstream peak temperature (Carpentier et al. 2017). In late summer and autumn, the stratification breaks as the reservoirs are drawn down through the spillway to provide flood storage capacity for the coming winter and spring precipitation. This leads to a well-mixed reservoir with downstream temperatures warmer than the natural regime. Such alterations in temperature regime, also termed as thermal pollution create challenging conditions for spawning and rearing of certain fish species and can be lethal for aquatic life (Olden and Naiman 2010).

The persistent thermal pollution from hydropower infrastructure worldwide, if left unaddressed, can potentially dwarf the benefits harnessed for renewable energy. According to the prediction from US Energy Information Administration, world’s energy demands will grow up by 50% from 2018 to 2050, mostly driven by steep rise in developing nations (U.S. Energy Information Administration 2019). This is proportionally increasing the installation of newer hydropower capacity in these countries. One of the striking examples is that of Laos which is aiming to become the “battery of Southeast Asia” by investing heavily in the hydropower dams across the nation (Rujivanarom 2019). While such a rise of new hydropower dams in emerging economies is inevitable, the only way to sustain the ecosystem while still generate clean energy is to improve their operational efficiency in terms of minimal impacts to the ecosystem.

### Need to improve hydropower efficiency

In contrast to developing nations, developed nations have saturated their dam installation capacity (Labadie 2004). As the escalating environmental impacts are being identified, the efforts have started shifting towards mitigation. The Federal Energy Regulatory Commission (FERC) in United States examines the environmental impacts and issues operational changes through 30- to 50-year licenses (Bednarek 2001). There have also been efforts to undam the rivers when the mitigation tolls are not enough. More than 1200 dams have been removed in the United States, especially in the past two decades (Bellmore et al. 2017). While dam removal has become commonplace to deal with aging and uneconomical dams, the resulting loss of reservoir habitat and movement of sediments can incur heavy costs to the ecology and environment (Stanley and Doyle 2003). Given the increasing need for clean and stable supply of baseload (Matek and Gawell 2015), removing the infrastructure would also be unfavorable for sustainable energy goals. From a logistical standpoint, the time and accrued cost of each dam removal would demand immense resources and a few centuries to remove all the dams the right way. As dams have become pervasive features of the river systems, continued improvement in the efficiency of dam operations is therefore the more pragmatic approach to maximize their benefits to humans and ecosystem.

Despite the recognized impact of dams on river’s thermal regime (Olden and Naiman 2010; Gu et al. 1999; Niemeyer et al. 2018; Rheinheimer et al. 2014), the quantitative effect of hydropower operations on downstream water temperature and the subsequent consequences on ecosystem have received little attention (Bonnema et al. submitted). Mitigation efforts to reduce thermal pollution from hydropower dams either focus on structural measures such as construction of selective withdrawal structures (Rheinheimer et al. 2014) or, by specifying required instream or minimum spillway flow downstream of the reservoir (Tharme 2003) based on an environmental flow assessment (King et al. 1999). The selective withdrawal outlets require additional construction and can be unviable for a reservoir due to the involved logistics and monetary constraints. Relying on environmental flows for controlling the downstream temperatures is prone to result in suboptimal conditions for the aquatic habitat particularly in conditions when inflow regime deviates from the climatology. Instead, a more dynamic scheme that considers inflow forecast information at short-term weather scale can guide the dam operator ahead of time on optimal operations for realizing ecologically safer downstream conditions (Ahmad and Hossain 2020).

Optimization of reservoir operations has been extensively studied for various operating objectives at short- and long-term operation scales (Labadie 2004; Yeh and Becker 1982; Barros et al. 2003; Ahmad et al. 2014). Multi-objective optimization for hydropower has been performed to satisfy other stakeholder benefits of flood control, water supply, irrigation and water quality (Le Ngo et al. 2007; Yazicigil et al. 1983; Shaw et al. 2017; Asadieh and Afshar 2019). Ahmad and Hossain (2020) optimized daily operations of two dams in US to maximize hydropower without compromising flood control. Jordan et al. (2012) presented optimization of turbine and bottom outlet operations for flood protection in a hydropower multi-reservoir system in Switzerland. Similar to flood control, maintaining a stable thermal regime also competes against the energy maximization objective as higher release or storage can significantly change downstream temperature. However, the inclusion of downstream river temperature as a constraint has not yet been explored or reported in published literature to the best of our knowledge.

### Need to model reservoir temperature

Incorporating water temperature as a constraint within an optimization scheme for hydropower generation requires quantitative relationship between the reservoir operations and changes in downstream thermal regime. There have been efforts to model the river temperature using deterministic and statistical models. Deterministic models, based on governing equations for heat transport, flow, and climatic conditions, do not explicitly include the reservoir operations as parameters for modeling temperature (Benyahya et al. 2007). Also, they typically require intensive hydrological and meteorological data input and computational effort in model building and calibration. Distributed river temperature models also exist that simulate river network by discretizing the river cell (Li et al. 2015; Yearsley 2012). Some of them often explicitly simulate reservoir’s thermal stratification by integrating land surface models (LSMs) with hydrodynamic models (Niemeyer et al. 2018; Buccola et al. 2016). Even complex three-dimensional models have been used such as by Jiang et al. (2018) to study thermal pollution in Lancang River using Delft3D-FLOW model. However, a major limitation with these complex models is the inability to integrate them with the hydropower optimization framework.

Another challenge towards temperature-constrained optimization is the dearth of in situ temperature measurements. The water temperatures in rivers are limited by sparse sampling in both space and time (Handcock et al. 2012). The scarcity of in situ temperature measurements is even more prominent in the developing nations that present major hurdles in building and validating the temperature models. Recent advancements in thermal infrared (TIR) remote sensing can quantify spatial and temporal patterns of surface water temperature at multiple spatial scales (Ling et al. 2017). This has been demonstrated by Bonnema et al. (submitted) where dry season water temperature cooling trends correlated with dam development in the Mekong basin, analyzed using 30 years of Landsat TIR observations. Thus, applications for ecologically sensitive hydropower optimization are better served by simpler river temperature model that can relate downstream temperature against decision variables for dam operations and global-scale satellite-derived temperature (where in situ data is scarce).

Only a few studies have explored simple regression models for stream temperature changes. Neumann et al. (2003) presented empirical model for daily maximum stream temperature in summers using average daily flow and air temperature as predictors. Mohseni et al. (1998) predicted weekly temperatures for fish habitat evaluation using nonlinear function of weekly air temperatures. The heat storage effects were considered by developing separate models for warming and cooling season. Benyahya et al. (2007) reviewed different regression models used for stream temperature. However, inclusion of reservoir operations in the regression model at daily time step has not yet been investigated in the literature. Because ecological impacts are more sensitive to changes in downstream temperature from natural thermal regime and not their absolute values, regression model offers an attractive alternative for the purpose.

**Fig. 1:**
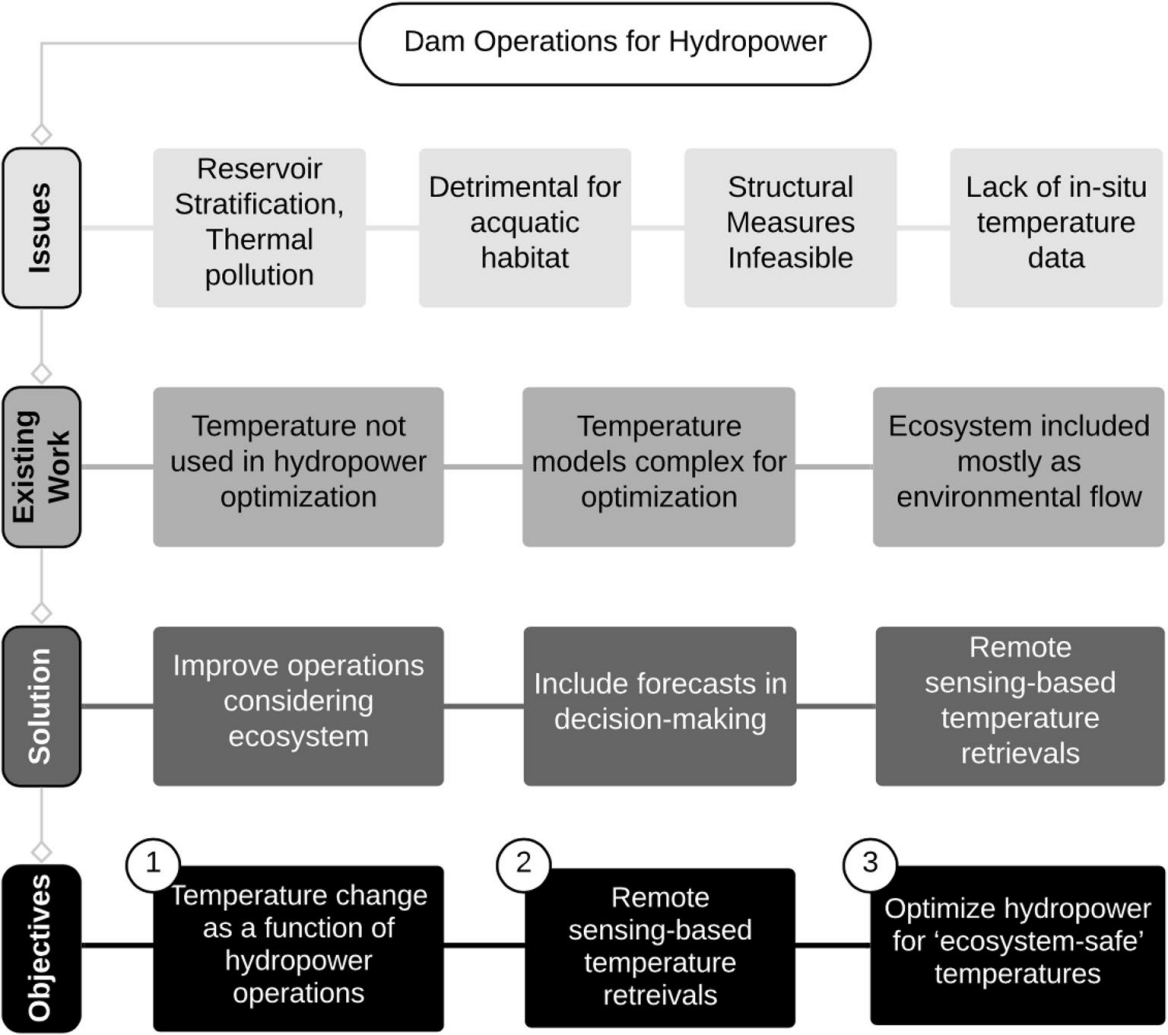
Pertinent issues with the current state of hydropower operations, brief summary of the existing literature and proposed solutions leading to the study objectives

The pertinent issues with the current state of hydropower operations, brief summary of the existing literature and proposed solutions leading to the objectives of this study are shown in Fig. 1.

### Study objectives

The goal of this study is first to tackle the challenges presented so far using the low hanging fruit of global weather forecasts from Numerical Weather Prediction (NWP) model. The use of NWP models for benefits to hydropower is well established (Ahmad and Hossain 2019a) and running operationally for a dam in US (http://depts.washington.edu/saswe/damdss/). The hydropower optimization is refined in this study by imposing temperature-based constraints to explore if hydropower benefits can still be realized while maintaining ecosystem thermal stability. The overarching question addressed here is: *can we generate more hydropower using weather forecasts while balancing ecosystem needs from a thermally stable regime standpoint*? The question is further broken down into tangible research objectives: (1) to understand downstream temperature change as a function of hydropower operations, (2) to develop a remote sensing-based approach for temperature modeling that can be used in developing nations, and (3) to optimize hydropower operations while ensuring ‘ecosystem-safe’ downstream water temperatures. The rest of the paper is organized as follows. In “Tools and datasets” section, the selected site and necessary datasets are described. This is followed by a description of the various methods used in “Methods” section. The case study results on demonstrating the eco-safe hydropower generation are presented in “Results” section, followed by discussion and concluding remarks in “Discussion” section.

## Tools and datasets

### Study site

**Fig. 2:**
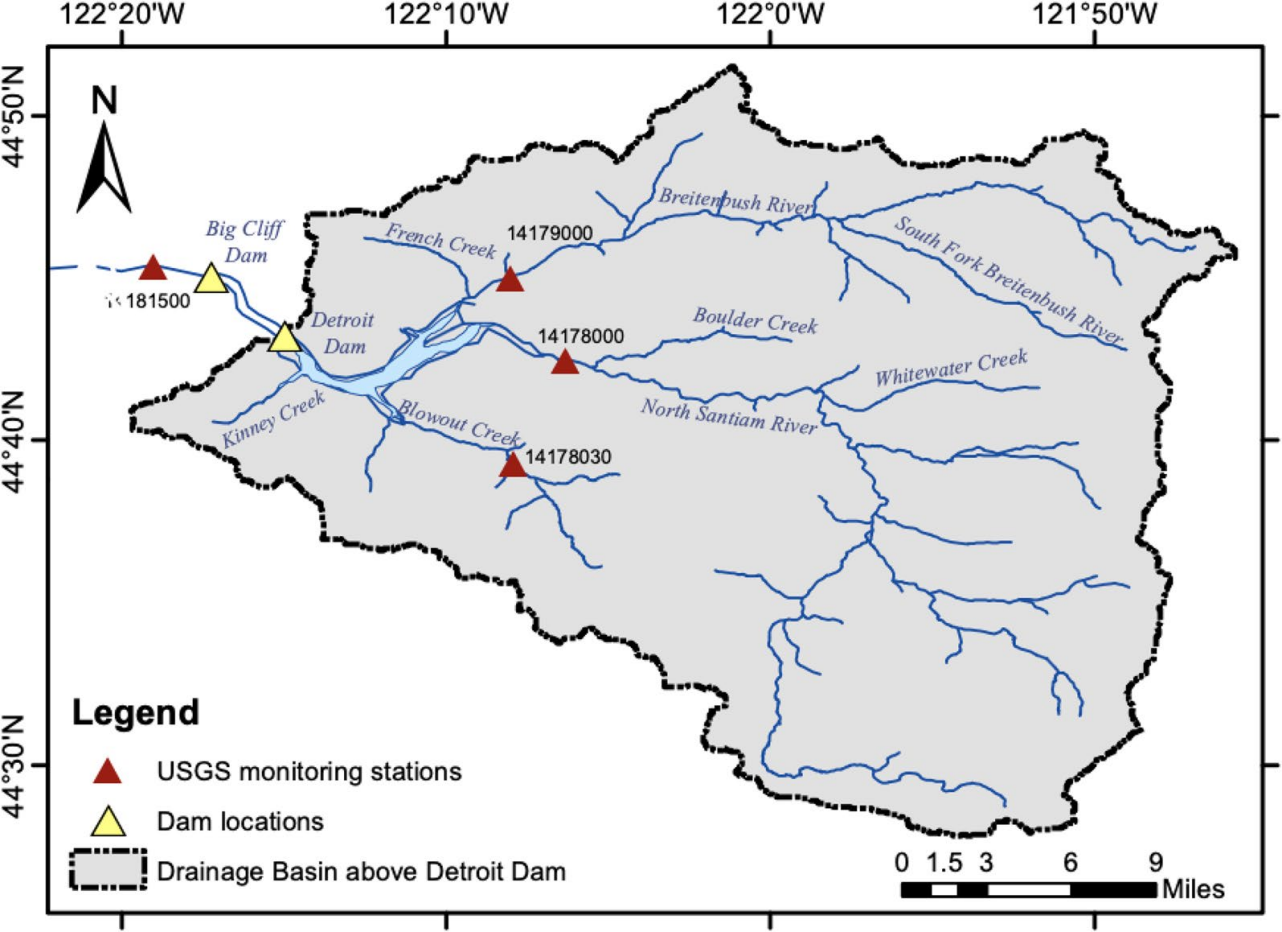
Drainage basin above Detroit dam (OR) and pertinent USGS monitoring stations used in the study

The Detroit Dam on the North Santiam River, Oregon (Fig. 2), controlled by U.S. Army Corps of Engineers (USACE), is authorized to provide flood control, hydroelectric power, navigation, and water in summer for irrigation and recreation. ﻿The reservoir is relatively small with a storage capacity of 455,000 ac-ft and storage to annual inflow ratio of 0.28. The powerhouse is designed for nameplate capacity of 100 megawatts (MW) with a hydraulic capacity of 5340 cfs. Located around three miles downstream of Detroit dam is the Big Cliff re-regulating dam with a small reservoir. The purpose is usually to smooth out the power generation release from the upstream Detroit dam and control fluctuations in downstream river level (Oregon Water Resources Department and U.S. Army Corps of Engineers 2012).

**Fig. 3:**
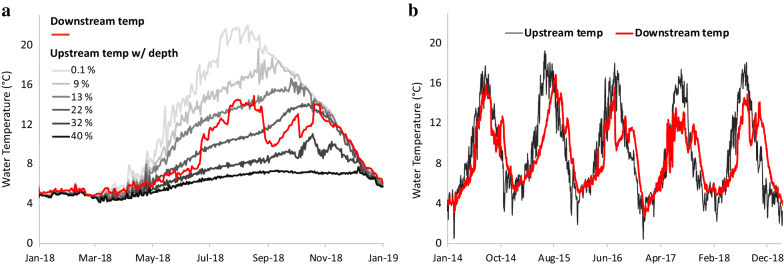
**a** Variation of upstream reservoir temperature with depth from spillway crest plotted as a percentage of maximum reservoir depth (440 ft) for Detroit dam (OR) in 2018. Downstream temperature is also plotted alongside; **b** time-series of stream temperatures upstream and downstream of dam showing alteration in downstream thermal regime, where flow-averaged temperature of upstream tributaries from USGS gages are used for upstream temperature [Source: USGS; USACE (2019)]

Since the commissioning of dam in 1953, the dam operations have changed the natural flow patterns in the basin to meet the authorized objectives. The changed flow patterns have also led to unintended consequences for the ecosystem and aquatic life (Oregon Department of Environmental Quality 2006). The variation in reservoir temperatures is shown in Fig. 3a where downstream temperatures usually correspond to the temperature of one of the pools in hypolimnion, depending on the penstock release. The reservoir stratification and bottom release of stored water have not only led to alterations in the downstream river temperature magnitudes but also in the timing of low and high temperature occurrences. As shown in Fig. 3b, higher stream temperatures that normally occur in July and August have been shifted towards September and October.

### Datasets—observed and forecast

To understand how the downstream temperature changes as a function of hydropower operations, in situ measured temperatures were obtained from U.S. Geological Survey (USGS) stations located on both the upstream tributaries and downstream river channel (Fig. 2). Flow-averaged temperatures were obtained from USGS stations on three rivers upstream of Detroit reservoir (44° 43′ N, 122° 15′ W). The downstream temperature station is located below the Big Cliff dam and accounts for regulation effects from both the dams. The upstream stations measure temperature of the top surface or epilimnion of the reservoir while the downstream stations represent average temperature of the downstream water column due to reduced tailwater stratification. The forecast meteorological fields were acquired from the NWP model of Global Forecast System (GFS) for forecasting reservoir inflow. The GFS fields were acquired at 0.5° resolution for 1–7 days lead-time with a 3-hourly temporal resolution. Air temperature was obtained from CPC Global Temperature data provided by the NOAA/OAR/ESRL PSD, Boulder (https://www.esrl.noaa.gov/psd/). The observed reservoir inflow and operations data were obtained from USACE (2019).

### Datasets—remote sensing

The primary data source for remote sensing-based water temperature estimation was a series of Landsat-7 ETM+ (Tier 1) satellite images. The TIR band (10.45 to 12.5 µm) is acquired at a resolution of 60 m. The image processing and temperature estimation analysis was performed in the cloud computing environment provided by Google Earth Engine (Gorelick et al. 2016).

**Fig. 4:**
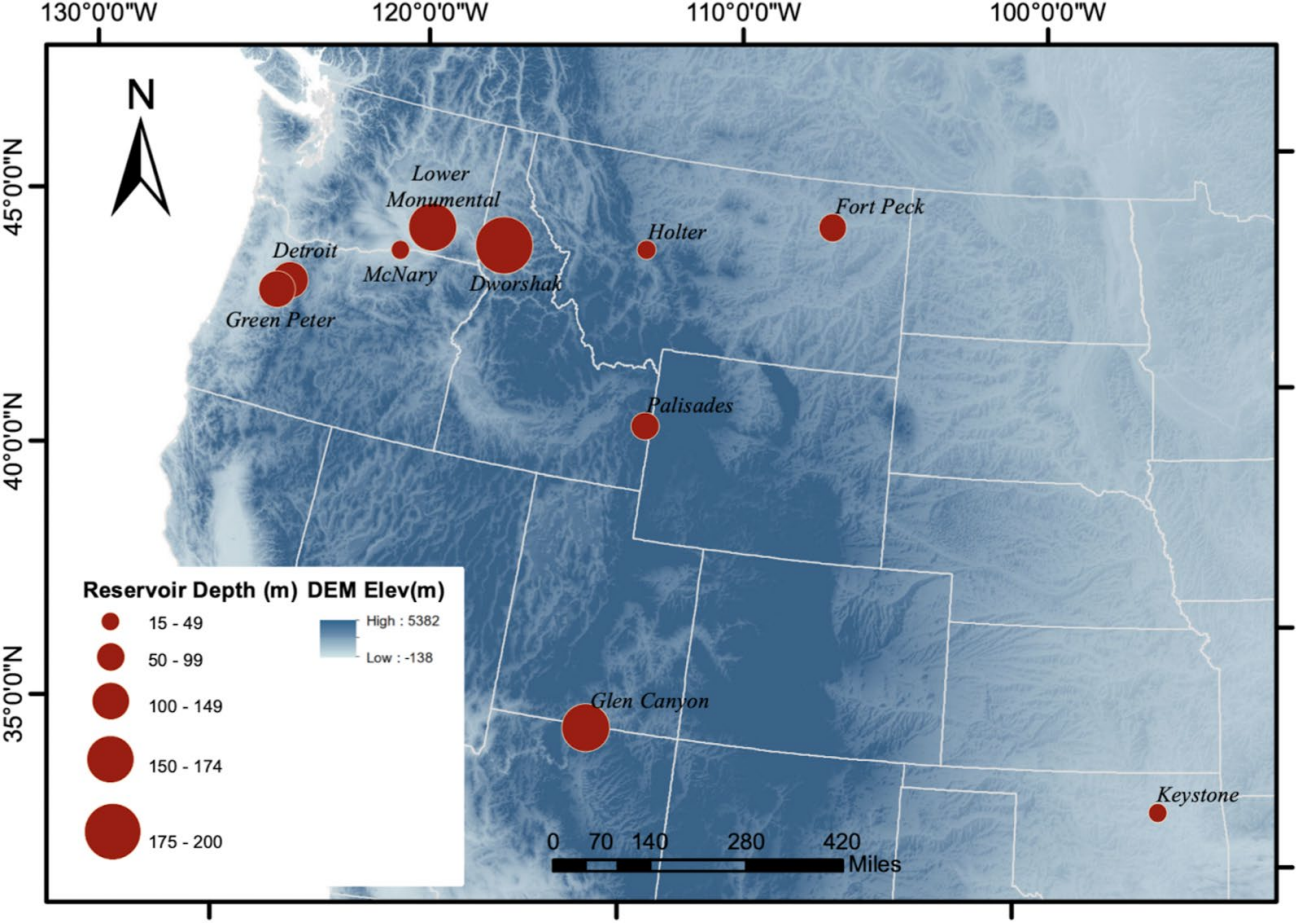
Selected dams for establishing the remote sensing-based temperature estimation. Markers are sized with their respective average reservoir depths

As the river channel downstream of Detroit dam is quite narrow, the pixels in TIR band acquired over water at 60 m possibly represent mixed pixels with a portion of reflectance contributed by surrounding land cover. Thus, ten dam sites with varying reservoir depths and downstream river width were chosen to explore the effect of pure water pixels in temperature extraction. The locations of selected dams and their average reservoir depths are shown in Fig. 4. Additional file 1: Table S1 summarizes the selected dams, their coordinates, approximate downstream river channel widths, respective Landsat-7 ETM+ scene path and row numbers, and USGS stations for upstream and downstream in situ temperature measurements.

## Methods

**Fig. 5:**
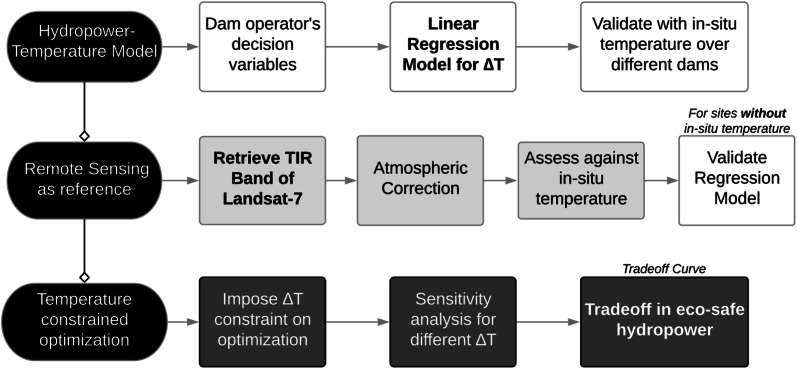
Experimental approach showing development of the temperature model, validation using remote sensing and its integration with the reservoir operations optimization to realize tradeoffs in ecosystem-safe hydropower generation

The study first establishes relationship between hydropower operations and temperature to be used for constraining the optimization problem. Remote sensing-based temperature estimation algorithm was established for validating the relationship in data-limited regions. Figure 5 summarizes the experimental approach followed to address the objectives.

### Modeling temperature–hydropower operations relationship

In order to understand the role of dam’s hydropower operations in modifying downstream river temperatures, we opted for a statistically based model that characterizes the statistical relationships between river temperature and decision variables for the dam operator. A simple statistical relationship between the reservoir operations and water temperatures also facilitates integration with the optimization model. We use the functional linear regression model relationship for this purpose.

For the linear regression relationship, daily difference between the upstream and downstream water temperatures was selected as the dependent variable. This is adequate because, firstly, the aquatic habitat is more susceptible to the relative difference between the downstream thermal regime and natural (upstream) conditions compared to their absolute values. Secondly, the statistical relationship is usually suited for comparative analysis and in predicting relative difference in modeled variable instead of its absolute value, which would require a more complex model (Yuba County Water Agency 2007). A set of candidate decision variables were selected as: (i) total release rate from reservoir, $R_{\text{t}} ,$ (ii) penstock release, $R_{\text{p}}$, (iii) turbine operating hours (captured in total hydropower generation, ${\text{HP}}$), (iv) reservoir forebay elevation, $E$, (v) inflow into reservoir, $I$, and (vi) air temperature, $T_{\text{a}}$. As tailwater elevation of the reservoir does not vary much with the tailrace discharge to significantly alter hydropower production, a constant value of 1200 ft was assumed based on the average value over past 10 years. The spillway release was obtained by subtracting the penstock release from the total discharge. The relationship between water and air temperature deviates from linearity for low (sub-zero) and high air temperatures (Mohseni et al. 1998) that necessitates a transformation function for air temperature to accurately capture the full variability. A logistic function was proposed by Mohseni et al. (1998) to describe the S-shaped air–water temperature relationship at weekly scale. We employ this function to transform the air temperature before feeding into the regression model:

1 $$T_{\text{a}}^{{\prime }} = \frac{\alpha }{{1 + e^{{\gamma \left( {\beta - T_{\text{a}} } \right)}} }},$$

where $T_{\text{a}}^{{\prime }}$ is the transformed temperature, the coefficient $\alpha$ represents the estimated maximum stream temperature, $T_{\text{a}}$ is the air temperature, $\gamma$ is a measure of the steepest slope of the function, and $\beta$ represents air temperature at inflection point of the S-shaped relationship (Mohseni et al. 1998). Buccola et al. (2016) implemented this approach for Detroit dam and obtained the fitting parameters for daily water temperature. The present study borrowed fitting parameter values, $\alpha = 18.08$, $\gamma = 0.10$ and $\beta = 20.42$ for North Santiam River at Boulder (USGS ID 1718000) from Buccola et al. (2016).

The decision variables were chosen such that the regression model can be used for predicting future temperature changes. The release rates, hydropower generation and elevation are outputs from reservoir operation model, reservoir inflow is derived from flow forecasting model (“Forecasting reservoir inflow” section) and air temperature can be obtained from NWP model forcings.

#### Seasonality in water temperature

In using the inherent relationships of different decision variables with the temperature change, seasonal variation in reservoir’s behavior is not modeled explicitly. The reservoir usually exhibits varying temperature signals based on changes in the stratification with seasonal temperature. Not accounting for seasonal stratification in the temperature model can induce seasonal bias in modeled temperature and poorer model performance. One way to indirectly account for the characteristic behavior across different seasons is to use a piecewise linear regression, fitting different relationships for different periods of the year. However, temperature data needs to be divided into separate chunks where the piecewise model might not result in representative slopes at the upper or lower ends of individual chunks (Mohseni et al. 1998). Time-varying coefficients can capture the variability over time (Li et al. 2014), however one drawback is the difficulty in interpreting the parameters in case of a multiple regression model.

The problem of seasonal variation and in particular, distinguishing the trend and cyclical movement components, has been dealt by economic analysts by performing adjustment for seasonal patterns within the regression model (Thomas and Wallis 1971). To perform this adjustment and capture the seasonal variation in water temperatures in addition to daily trends, we include additional seasonal dummy variables in the model. The seasonal dummies are a function of frequency at which seasonal behavior is prominent for the reservoir in consideration. The seasonality that is explicitly modeled here captures the deterministic seasonal processes and is usually termed as deterministic seasonality. Mathematically, let $s$ be the seasonal frequency (dividing year into $s$ different periods) and let $D_{1t} , D_{2t} , \ldots D_{st}$ be seasonal dummy variables for any particular day $t$, corresponding to periods $1, 2 \ldots , s$. For a selected day $t$, one of the seasonal dummies $D_{it}$ equal 1, while all the others equal 0,

2 $$D_{it} = \left\{ {\begin{array}{ll} {1, \quad {\text{if}}\;{\text{observation}}\;{\text{at}}\;{\text{time}} \;t\;{\text{is}} \;{\text{in}}\; i{\text{th}}\;{\text{period}}} \\ {0, \quad {\text{otherwise}}} \\ \end{array} } \right..$$

Monthly timestep was chosen here as the seasonal frequency for modeling reservoir’s seasonal behavior. Hence, the deterministic seasonality, $S_{t}$, can be expressed as a linear function of dummy variables,

3a $$S_{t} = \left\{ {\begin{array}{ll} {\theta_{1} , \quad {\text{if}}\;\;t = {\text{Jan}}} \\ {\theta_{2} , \quad{\text{if}}\;\;t = {\text{Feb}}} \\ \vdots \\ {\theta_{12} , \quad {\text{if}}\;\;t = {\text{Dec}}} \\ \end{array} } \right.$$

3b $$S_{t} = \mathop \sum \limits_{i = 1}^{s} \theta_{i} D_{it}$$

where $\theta_{i}$ are the regression coefficients for each dummy variable and $s$ = 12 for the monthly frequency.

#### Formulation of regression model

Apart from the seasonal components, optimal set of decision variables to capture the daily trends were selected based on a sensitivity analysis. The analysis was based on indicator metrics of correlation coefficient, Akaike’s Information Criteria (AIC) and mean absolute error (MAE) of the model. AIC tries to maximize the explained variance in predictors while also minimizing the variance of the resulting estimates by limiting the number of coefficients (Neumann et al. 2003). The least squares regression model can then be formulated as,

4 $$y_{t} = \alpha + \mathop \sum \limits_{k = 1}^{n} \beta_{k} P_{k} + \mathop \sum \limits_{i = 1}^{s - 1} \theta_{i} D_{it} + \varepsilon_{t} ,$$

where $t$ spans the days of year, $y_{t}$ is the modeled temperature difference between upstream and downstream, $\alpha$ is the intercept, $\beta_{k}$ is the regression coefficient for the $k{\text{th}}$ predictor variable $P_{k}$ ($k = 1,2 \ldots n$), where $n$ is the total number of selected predictors, seasonal frequency $s = 12$, and $\varepsilon_{t}$ accounts for unexplained variation in modeled temperature for time $t$. It should be noted that the regression is performed by omitting one of the monthly dummy variables (e.g., December), for they would be collinear and redundant.

### Water temperature from remote sensing

**Fig. 6:**
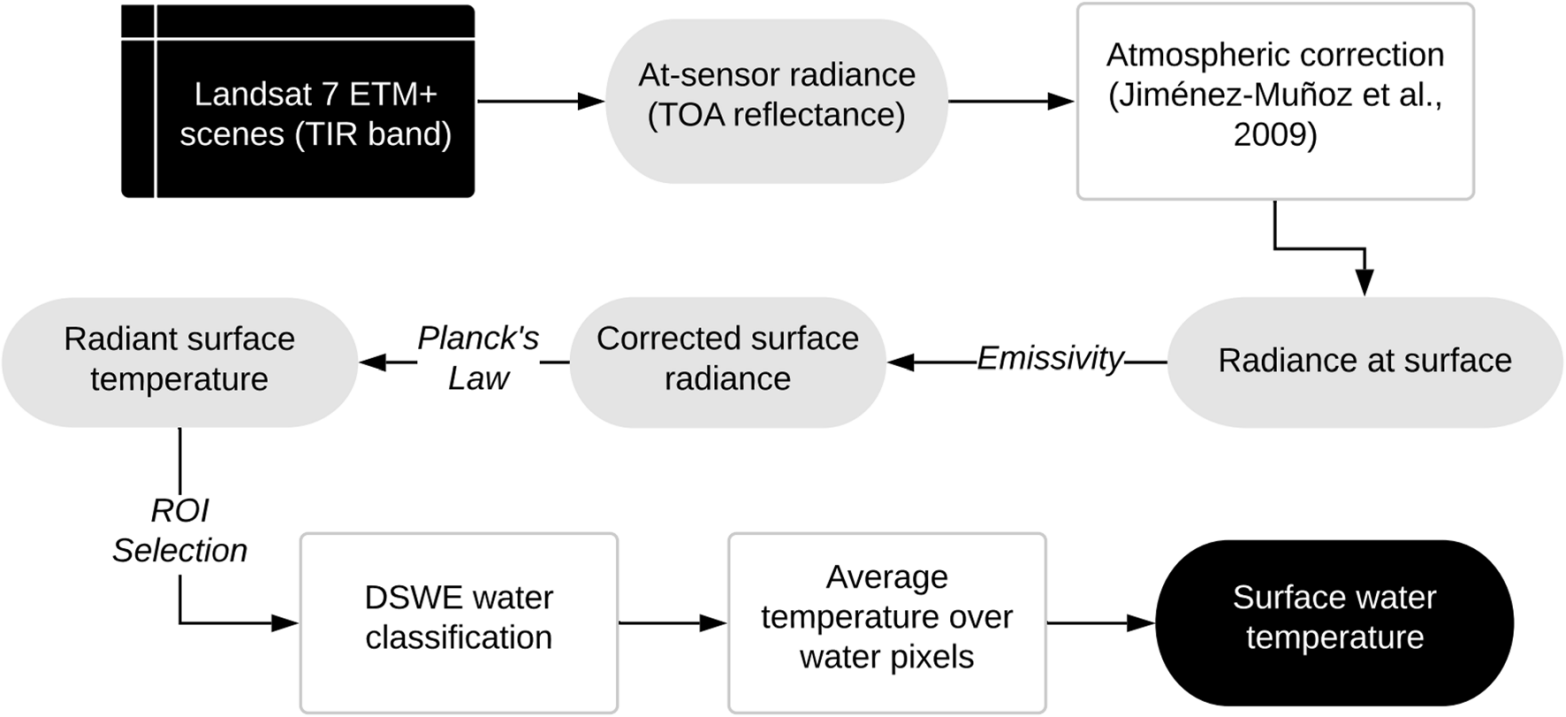
Single channel (SC) algorithm using Landsat ETM+ for estimating water temperature upstream and downstream of dams

Remote sensing was used to obtain water temperature so that the technique can serve as a potential reference when in situ temperature data is scarce or absent. From the decade-long record provided by Landsat-7 mission, the thermal infrared (TIR) band was used to extract the water temperature using single channel (SC) algorithm (Jiménez-Muñoz and Sobrino 2003; Jiménez-Muñoz et al. 2008) both upstream and downstream of the dam. The temperature estimation algorithm is shown schematically in Fig. 6.

Top-of-atmosphere (TOA) reflectance from TIR band of cloud-free Landsat 7 scenes was atmospherically corrected. The correction procedure used information about upwelling and downwelling radiances and atmospheric transmissivity, estimated using atmospheric functions (AFs). We used coefficients derived by Jiménez-Muñoz et al. (2008) to relate AFs and water vapor content for the operative SC algorithm. The ground leaving TIR radiance was corrected with an emissivity of 0.99 for distilled water (Handcock et al. 2006). The resulting corrected radiance led to the calculation of radiant surface temperature using Planck’s Law. Water pixels were classified using Dynamic Surface Water Extent (DSWE) algorithm within regions of interest (ROIs) containing USGS measurement stations (Jones 2015). Pixels with high confidence were retained for averaging surface temperature estimate from low-gain TIR Band (B6 VCID 1) of the processed Landsat 7 image. Averaging the temperature retrievals also help minimize the effect of any possible contamination of reflectance from the surface due to factors such as eutrophication or presence of vegetation on the water surface.

### Ecosystem-sensitive hydropower via multi-objective optimization

We modeled dam operations at daily time step using water balance approach to optimize for reservoir releases leading to optimal flow designs (see Fig. 5). The reservoir’s actual bathymetry was factored in the model using a storage–elevation relationship derived for the reservoir. Release decisions were optimized using a multi-objective optimization model. The primary objective was to maximize hydropower generation from powerplant over an optimization horizon of 7 days.

5 $$\hbox{max} f_{1} \left( {\text{MW}} \right) = \mathop \sum \limits_{t} \varepsilon \cdot \Delta t_{\text{turb}} \cdot \left( {{\text{HF}}_{t} - {\text{HT}}_{t} } \right) \cdot R_{p,t} .$$

The secondary objective was set to minimize a penalty cost function that accounts for the long-term effects of the release decisions over the short-term optimization horizon. The penalty function is quantified based on the deviation of reservoir storage $S$ from the rule curve-specified level ${\text{RC}}$, which is representative of the long-term optimal state of reservoir under climatological flow regime:

6 $$\hbox{min} f_{2} \left( {\text{acft}} \right) = \mathop \sum \limits_{t = T}^{7} \left| {S_{t} - {\text{RC}}_{t} } \right|,$$

where deviation is considered starting $T{\text{th}}$ day of the 7-day optimization horizon. Under normal flow circumstances, $T$ was set to two to consider last six days of the horizon for calculating the deviation. During high inflow periods, where the forecasted inflow exceeds the turbine capacity, deviation was calculated only over the last two days to slacken the penalty function and give more room for controlling the high inflow event.

The optimization problem involves mutually conflicting objectives where it would be impossible to realize a single release schedule that satisfied both of them perfectly. Thus, a balance in tradeoff solutions was achieved using Pareto optimality. An optimal solution that gives equal weightage to both the objectives was selected on non-dominated set of solutions of a Pareto front. The cost to ecosystem was considered in terms of change in riverine thermal regime while designing the optimal releases. The hydropower–temperature relationship was incorporated to impose additional constraint on the optimization model to guide the release decisions. The modeled temperature difference between upstream and downstream reaches was limited to a selected minimum and maximum threshold,

7 $$\Delta T_{\text{min} } \le \Delta T \le \Delta T_{\text{max} } .$$

The temperature-driven constraints in Eq. (7) form the essence of realizing ecosystem-safe hydropower using weather forecasts from thermally stable regime viewpoint. The prescribed window between $\Delta T_{\text{min} }$ and $\Delta T_{\text{max} }$ determines the level of ‘safety’ that the optimization aims to attain. Other constraints pertaining to dam safety, reservoir storage, and release rates were imposed based on the physical and operational limits of the reservoir. Readers are referred to Ahmad and Hossain (2020) for detailed formulation of the constraints. The reservoir operations were modeled using a water balance approach, where the amount of water in reservoir $S_{t}$ on day $t$ of the optimization horizon is a function of storage on the previous day $\left( {t {-} 1} \right)$ and the inflows, losses and releases on the current day:

8 $$S_{t} = S_{t - 1} + \delta \left( {I_{t} - L_{t} - R_{t} } \right),$$

where $\delta$ is a constant to extrapolate flow rates into daily volume units while assuming a constant flow within each day. As the optimization is performed at daily time steps, storage losses $L_{t}$ due to evaporation and seepage were ignored. The optimization was carried out using the Non-dominated Sorting Genetic Algorithm (NSGA-II) (Deb et al. 2002).

#### Forecasting reservoir inflow

For the inputs to the reservoir optimization model, weather-scale inflow forecasts were modeled using a machine learning technique to ensure high skill with computational efficiency in processing. The forecasting was based on a feedforward artificial neural network (ANN) involving input, hidden and output layers, as established in our earlier work (Ahmad and Hossain 2019b) to be valuable and skillful over multiple reservoirs in US. Forecast fields from GFS model were inputs to a three-layered ANN model along with antecedent hydrometeorological conditions of precipitation, temperature, basin’s runoff and baseflow. Consecutive daily ANN models were used to result forecast streamflow for 7 days in future. Our previous study (Ahmad and Hossain 2019b) describes the model development and predictor selection in more detail. Training was performed using Levenberg–Marquardt method and early stopped training (STA) was incorporated to avoid overfitting and lack of generalization.

#### Sensitivity to allowable change in temperature

The objective concerning hydropower generation demands larger storage and higher releases through the penstocks. This is likely in conflict with the goal of attaining a stable thermal regime, as larger reservoir storage intensifies stratification leading to larger temperature differences. Also, higher penstock releases end up cooling downstream reaches. We performed a sensitivity analysis to investigate the amount of hydropower benefit realized by imposing constraints of varying degrees of allowable change in temperature between upstream and downstream rivers. Allowable temperature-difference ($\Delta T_{\text{allow}}$) windows ranging from 1 to 6 °C of change were imposed as constraint to the objective of hydropower maximization for multiple years. Oregon Department of Environmental Quality (ODEQ) has prescribed Total Maximum Daily Loads (TMDLs) for temperature to ensure river does not exceed water quality criteria considering pertinent fish uses (Oregon Department of Environmental Quality 2006). The choice for $\Delta T_{\text{allow}}$ windows was driven by the observed deviations in downstream thermal regime from the prescribed ODEQ limits over the past decade. The selected windows signify resilience of the downstream ecosystem in response to hydropower operations. Moreover, the approach, by considering different $\Delta T_{\text{allow}}$ possibilities, is also able to study system’s resilience against future alterations in stream temperature due to climate change impacts.

#### Designing adaptive release policy

The sensitivity analysis focused on adhering to the natural thermal regime upstream of the dam. With changes in climate leading to larger temperature anomalies from the historical average, the upstream temperatures can render suboptimal for the habitat downstream of the dam. Here, we explore a more holistic approach to designing operations by considering specific ecosystem’s biodiversity and tolerance level of aquatic species to thermal instability, as informed by dam’s pre-existing biology. For the North Santiam River, regulated by Detroit dam, the most sensitive beneficial uses of the river include Salmonid fish spawning and rearing, and anadromous fish passage. Biologically based numeric criteria have been prescribed under TMDL for each season to meet the critical downstream uses (Oregon Department of Environmental Quality 2006; National Marine Fisheries Service 2008). The criteria, summarized in Table 1, are expressed as a 7-day moving average of daily maximum temperature.

**Table 1:** Biologically based numeric criteria prescribed under TMDL for North Santiam Subbasin of Detroit dam

Season	Downstream use	7-day average temperature criteria (°C)
September 1–June 30	Salmon spawning	12.8
Summer (July 1–August 31)	Salmon and steelhead rearing	17.8

We used these criteria to frame the multi-objective optimization for release decisions that adapt to the downstream habitat uses while still maximizing hydropower. Instead of temperature difference, the optimization framework now constrains the average of absolute downstream temperature over 7-day horizon within the required criteria for the respective season. Thus, the constraint in Eq. (7) is modified as,

9 $${\text{average }}(T_{{{\text{dn}},t}} ) \le T_{\text{target}} ,$$

where $T_{{{\text{dn}},t}}$ is the downstream temperature for $t = 1, 2 \ldots 7$ days and $T_{\text{target}}$ represents the biological criteria.

#### Evaluation of optimal decisions

We evaluated the results of ecosystem-safe reservoir releases that concurrently improve hydropower generation by comparing with benchmark operations for multiple years. Two benchmark scenarios were incorporated: (i) *business*-*as*-*usual (BAU)* based on the actual operations of reservoir under observed conditions, (ii) *climatological baseline* (CLB) that uses climatological flow instead of ANN-based forecasts to perform the multi-objective optimization and derive optimal reservoir release. No temperature constraint is imposed on optimization with climatological flows. BAU allows assessment of the degree of improvement possible with the proposed eco-safe optimization concept over the real-world operations. On the other hand, CLB provides a realistic and fair benchmark where the rules are derived under the same framework as used for inflow forecast-based optimization, which might not hold for BAU. Comparison with CLB explains the significance of using forecast information and imposing temperature constraints on the optimization model.

We used 50 years of reservoir inflow to derive the climatology which was then used to perform the hydropower optimization over 5 years of different inflow regimes without imposing any temperature constraint (Additional file 1: Fig. S1). The daily and annual inflow variations over the selected years are shown in Additional file 1: Fig. S2. Improvement in energy over the two benchmark scenarios in different years led to derivation of a tradeoff curve. The curve signifies mutual conflict between the possible hydropower improvement using weather forecasts and degree of thermal stability in downstream waters.

## Results

### Quantifying hydropower–temperature relationship

To quantify associations between change in thermal regime downstream of dam and hydropower operations, functional linear regression model was developed for Detroit dam. A stepwise procedure was followed for selecting the most optimal set of inputs to the model. The dependent variable was regressed against sequential combination of input variables over 5 years of daily data (2011–2015). Table 2 summarizes the indicator metrics of correlation coefficient, AIC and MAE for different models used in the stepwise regression procedure, underscoring the predictive skill in each of the inputs.

**Table 2:** Indicator metrics for models with different candidate predictors in the stepwise regression procedure (refer to “Modeling temperature-hydropower operations relationship” section for notations)

Model predictors	Correlation coeff. (*R*)	MAE (°C)	AIC
$R_{\text{p}}$	0.19	1.58	7624
$R_{\text{p}} ,{\text{HP}}$	0.45	1.41	7282
$R_{\text{p}} ,{\text{HP}},R_{\text{t}}$	0.47	1.41	7252
$R_{\text{p}} ,{\text{HP}},R_{\text{t}} ,E,I$	0.51	1.39	7179
$R_{\text{p}} ,{\text{HP}},R_{\text{t}} ,T_{\text{a}}^{{\prime }}$	0.58	1.37	6983
$T_{\text{a}}^{{\prime }}$	0.49	1.48	7204
$R_{\text{p}} ,{\text{HP}},R_{\text{t}} ,E,I,T_{\text{a}}^{{\prime }}$	0.59	1.36	6937
$D_{i}$ ($i$ = 1, 2,…12)	0.71	1.12	6476
$R_{\text{p}} ,{\text{HP}},R_{\text{t}} ,E,I,T_{\text{a}}^{{\prime }} ,D_{i}$	0.82	0.93	5737

The effect of including seasonality in the regression model via seasonal dummy variables is shown in Table 2 by the significant reduction in MAE and AIC of the resulting model. Air temperature, $T_{a}^{{\prime }}$ is transformed using the logistic function as described in “Modeling temperature-hydropower operations relationship” section. The statistical significance of each predictor was ensured with their *P* values consistently less than the 95% confidence $\alpha$ level of 0.05, except for the inflow. The resulting coefficients in the final regression model and their respective *P* values are shown in Table 3.

**Table 3:** Regression coefficients and statistical significance (*P* values) of the selected predictors

Predictor	$R_{\text{p}}$	${\text{HP}}$	$R_{\text{t}}$	$E$	$I$	$T_{\text{a}}^{\prime }$
Coefficient	− 0.0017	0.0041	− 2.2e−4	− 0.0038	− 2.9e−5	0.82
*P* value	2.1e−8	3.7e−13	4.0e−11	0.02	0.06	0.00

The coefficients of the regression model indicate the sensitivity of downstream temperature change to each of the independent predictors. For instance, difference between upstream and downstream water temperatures can go down by one degree decrease on a penstock release of ~ 600 cfs and can increase by one degree on an increase in air temperature of 1.3 °C. The positive sign on the coefficient for hydropower (owing to negative signs on penstock release and reservoir level coefficients) depicts the contrasting effect where a larger generation leads to higher difference between upstream and downstream water temperatures.

**Fig. 7:**
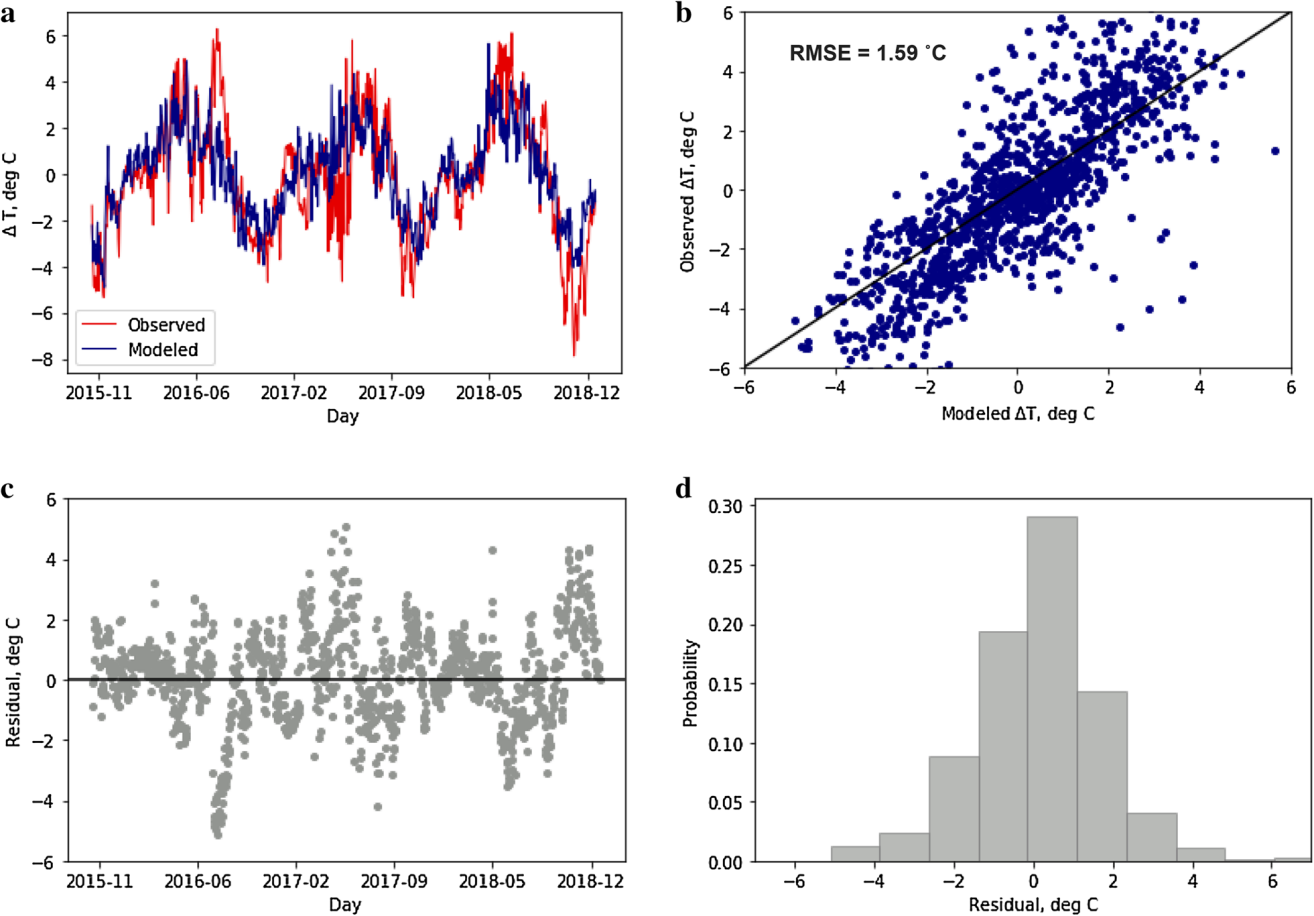
Performance assessment of the regression model for temperature change between upstream and downstream reaches: **a** time-series of observed and modeled variable, **b** scatter plot for the same, **c** time-series of the residuals in the modeled variable and **d** PDF of the residuals

The selected model was then validated over 3 years (2016–2018). Observed and modeled changes in temperatures are compared in Fig. 7a, b. Time-series plot of the residuals and their probability distribution function (PDF) are shown in Fig. 7c, d. The model is able to capture peaks and lows in temperature change and the residuals are mostly centered around 0 °C. The functional regression is able to explain 64% variance in temperature change as a function dam operations and seasonal dummy variables. Predictions from this temperature-hydropower model formed the basis for establishing remote sensing-based temperature estimation described next as well as for the multi-objective optimization model.

### Reservoir temperature from satellite remote sensing

**Fig. 8:**
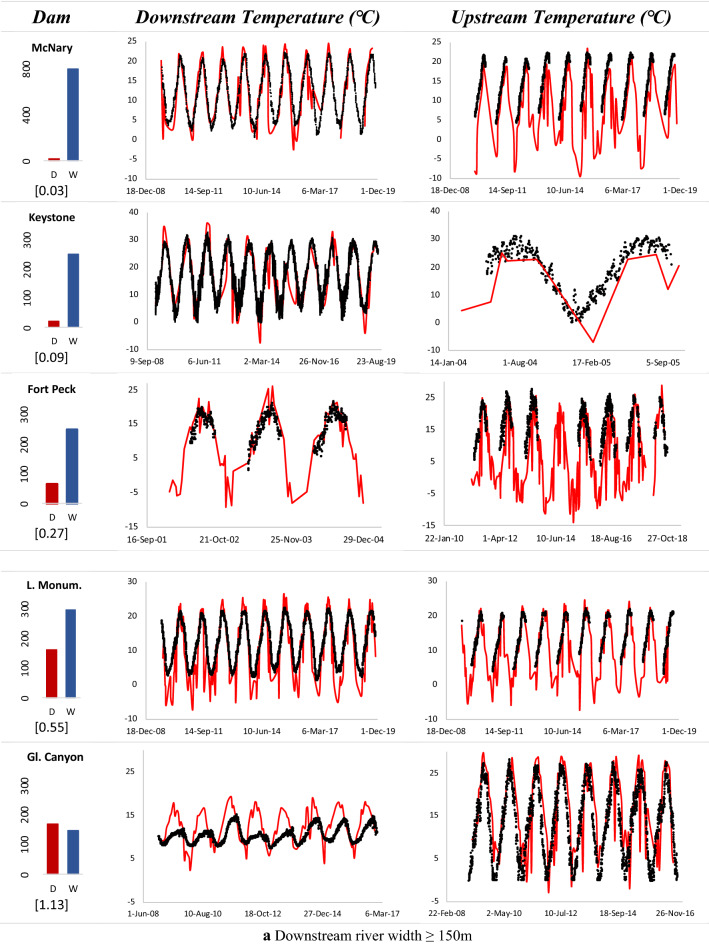

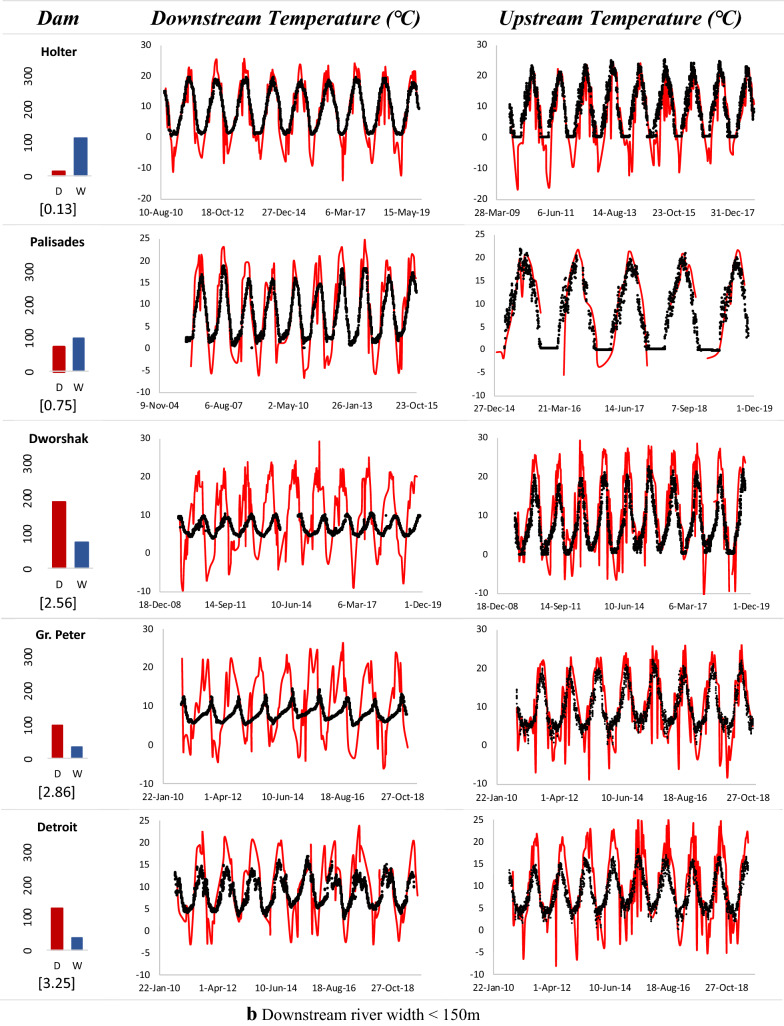
Time-series of remote sensing-based temperatures (red), compared with USGS in situ measurements (black) upstream and downstream of dams with **a***W* ≥ 150 m, and **b***W* < 150 m. The average reservoir depth (*D*) and downstream river width (*W*) in meters as well as D/W ratio (in square brackets) for each dam are shown alongside

The Landsat 7 satellite imagery was utilized to extract the water surface temperature along the reaches upstream and downstream of the dam. As the channel width downstream of the dam is critical in acquiring pure water pixels for temperature extraction, the SC algorithm was applied to multiple dams with varying river widths. Figure 8 shows the extracted remote sensing-based temperatures and qualitatively compared with the USGS in situ measurements both upstream and downstream of the dams.

The results suggest that for downstream reaches of rivers with depth-to-width ratio of less than or close to one and a width (*W*) of at least 150 m, both the upstream and downstream temperature estimates from remote sensing match well in terms of capturing variations and peaks when compared with in situ measurements.

**Fig. 9:**
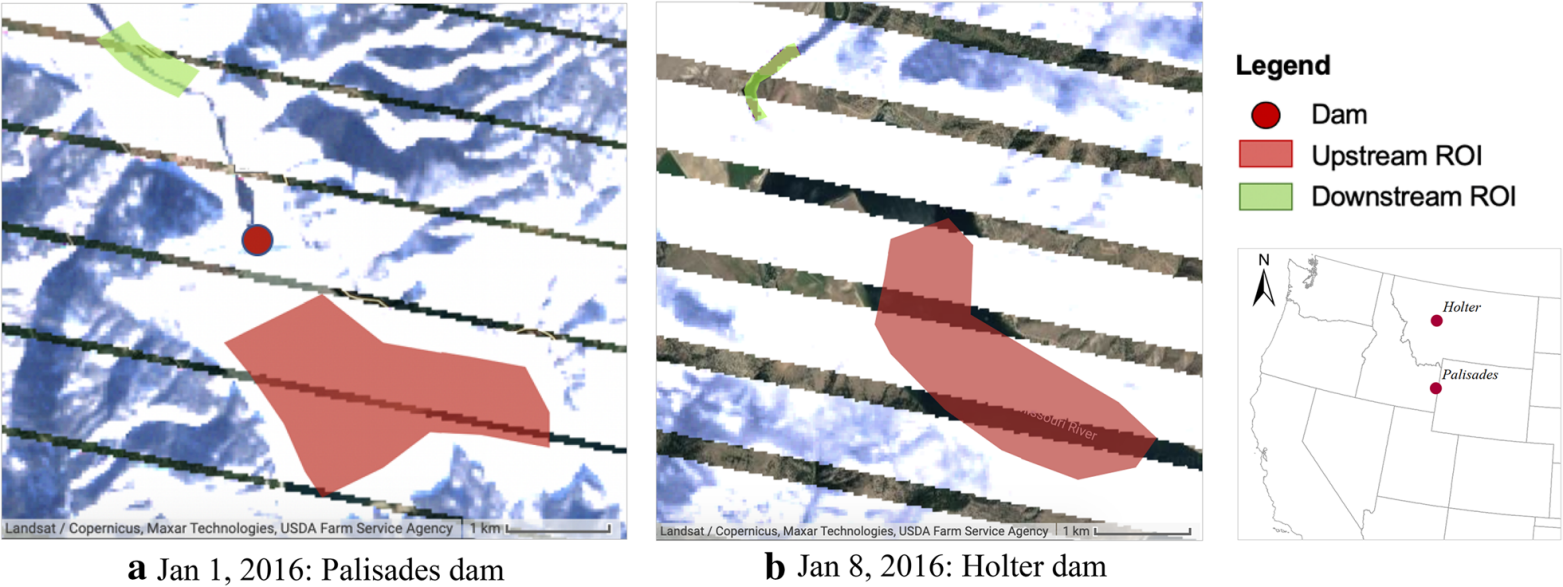
Landsat ETM+ images showing a sheet of ice forming on top of reservoir surface during winter season, resulting in sub-zero surface radiant temperatures for two dams. Green and red polygons (regions of interest; ROI) were used for obtaining average temperatures downstream and upstream, respectively

In general, when downstream channels entail more than two water-only pixels in TIR band (*W* ≥ 120 m), averaging over them results in improved estimates. Shallower reservoirs, on the other hand, have weaker stratification where surface closely represents the temperature of released water from deeper pools. The extracted temperatures were usually lower than the observed values across all the dams during winter season. As the TIR band provides measurement of radiant temperature at the surface or ‘skin’ layer of water (approximately top 10 cm), it is not representative of the kinetic or bulk temperature along the water column as measured by in situ sensor (Handcock et al. 2012). The difference in the two temperatures especially escalates during winter regimes where a sheet of ice forms on the surface, shielding the water below from dropping to sub-zero temperatures. Cloud interference was also a prominent issue during winters causing discrepancies therein. An example is shown in Fig. 9 for two dams.

For dams such as Detroit, Dworshak and Green Peter with narrower river channels and less than a couple pixels covering the river width, overestimation in peak temperatures was observed due to the issue of mixed pixels. For deeper reservoirs of Green Peter, Dworshak and Glen Canyon dams, remote sensing-based estimations exhibit rapid warming during pre-peak periods, reaching the peaks earlier than in situ measurements. This, in fact, is an artifact of the intensified stratification across the deeper reservoir pools, leading to warmer radiant surface temperature as compared to colder kinetic temperatures represented by in situ gages. The upstream reaches revealed better performance than their downstream counterparts across all the dams due to larger water area for averaging and a greater number of ‘pure’ pixels.

### Tradeoffs in hydropower generation while maintaining thermally stable regime

**Fig. 10:**
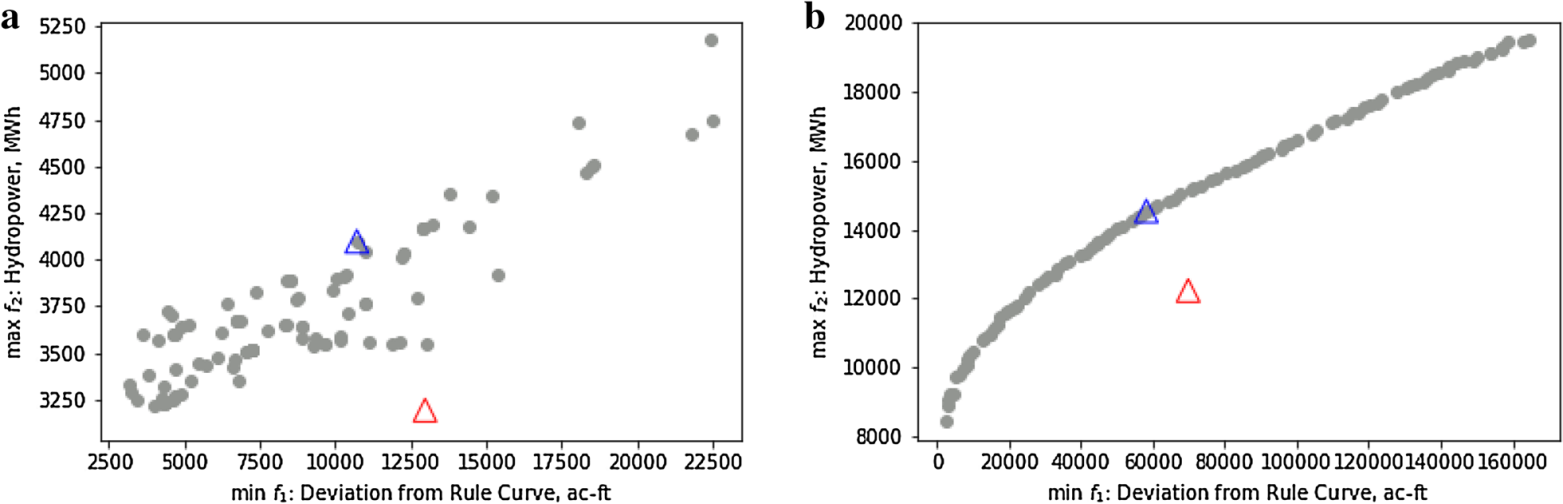
Sample Pareto frontiers between hydropower generations and storage deviation from rule curve, depicting the optimal release decisions for **a** 5 Jan 2014 (wet year) and **b** 4 March 2016 (relatively drier year). Blue triangle represents the selected solution for carrying out sensitivity analysis while red triangle is the location of respective objectives from BAU scenario

Based on the short-term inflow forecasts obtained from ANN model, the hydropower operations were optimized with varying temperature change constraints. The analysis was first performed over 2 years with different climate regimes: (i) dry year (below-average annual river discharge), and (ii) wet year (above-average annual river discharge). The Pareto frontier from the multi-objective optimization between hydropower maximization and storage deviation minimization during two different seasons is shown in Fig. 10. A sample solution, shown with blue triangle, is selected on the front to perform the sensitivity analysis and obtain the tradeoffs.

**Fig. 11:**
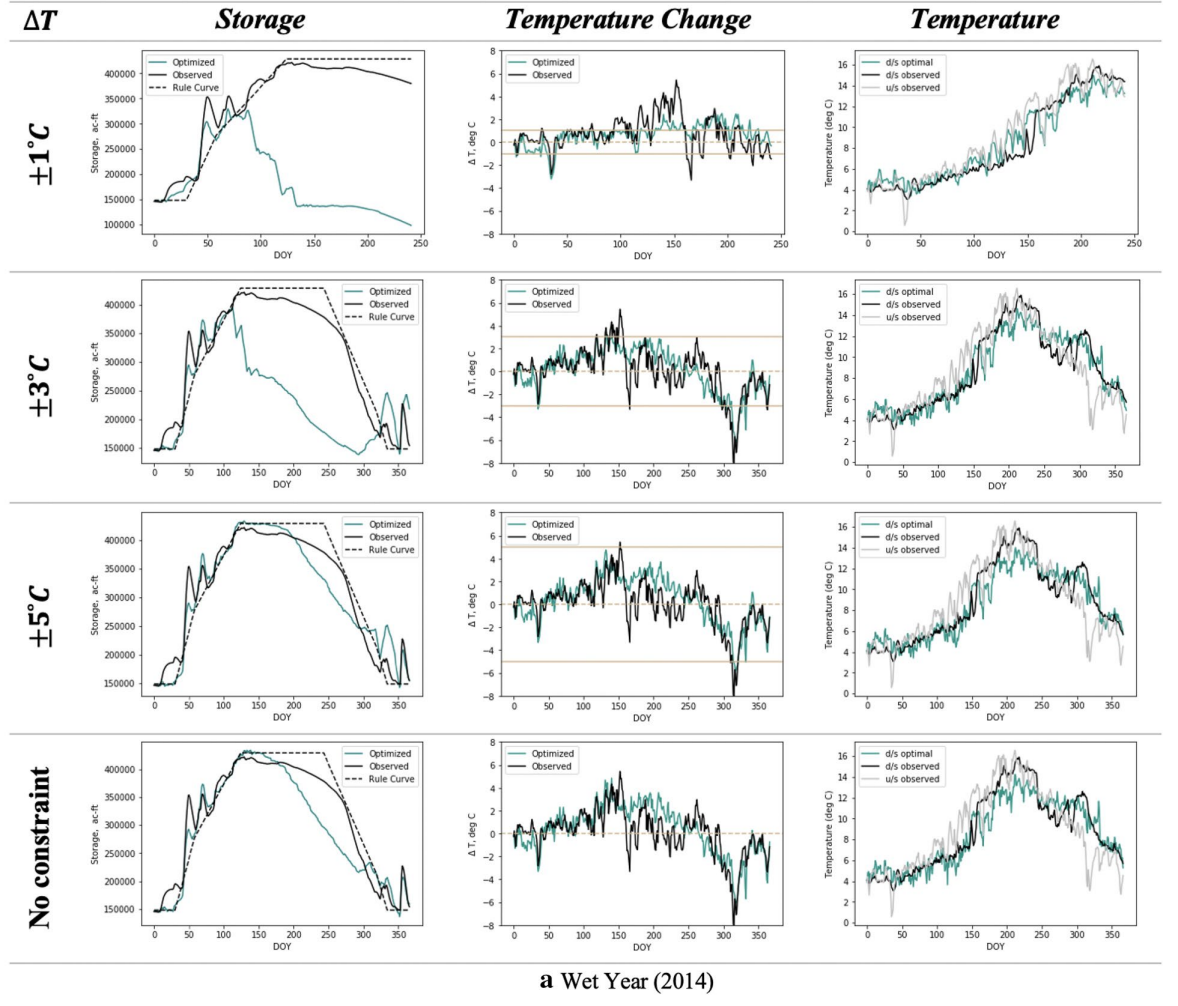

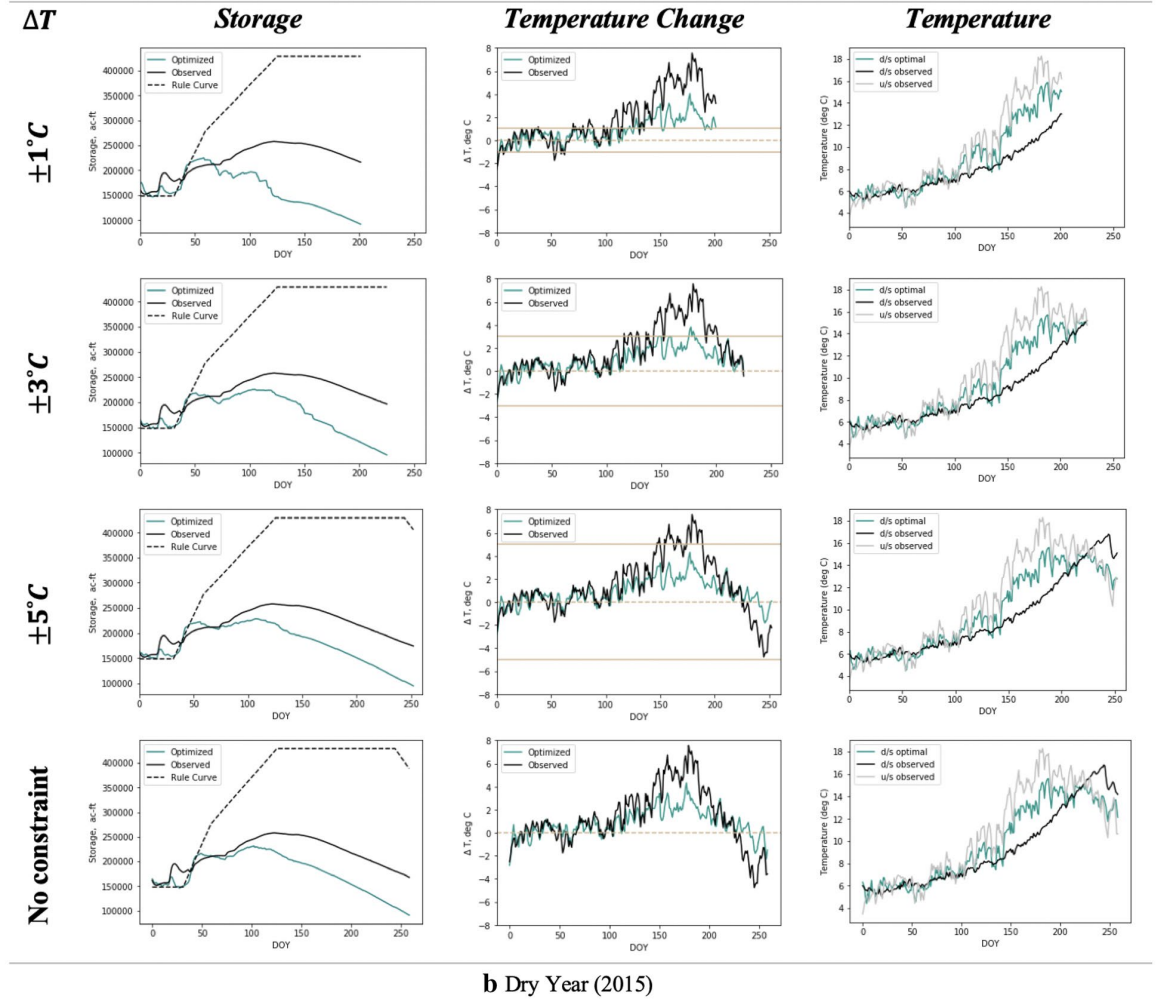
**a** Optimal reservoir states and downstream temperatures for different allowable temperature change scenarios over wet (high flow) year. Optimal downstream temperatures (third column) are derived from the respective optimal temperature changes (second column). **b** Same as Fig. 10a, but for dry (low flow) year of 2015

The resulting pareto optimal solutions consistently overperformed BAU scenario in terms of the considered objectives over different seasons. The selected solution on this front seeks to concurrently balance the objective of hydropower generation and penalty for deviation from the rule curve. The optimal reservoir states and corresponding downstream temperatures for different allowable temperature change scenarios are shown in Fig. 11.

To constrain the downstream temperature change within the allowable range, the reservoir has to be lowered based on the hydropower–temperature relationship. This leads to lower storage levels compared to the observed scenario as constraints became more stringent. Over the wetter year of 2014, the optimal downstream temperatures mimicked the upstream regime for almost all the allowable ∆*T* constraints. In the most stringent constraint scenario of 1 °C of allowable change (for a very sensitive or weak downstream ecosystem), the prescribed release policy led to rapid drawdown of the reservoir with levels reaching the minimum storage bound. This resulted in no feasible solution by the month of September (Fig. 11a). For the drought year of 2015, optimization ceased with infeasible solutions by July and August for all the scenarios as the reservoir was not able to recover due to low inflow volumes (Fig. 11b).

The hydropower benefits for each scenario, obtained in terms of improvement in energy generation over the benchmark of CLB, are shown in Table 4 for the year 2014. The benefits for the dry year 2015 were not quantified here for assessment as only a portion of the year was optimized. The analysis highlights the tradeoff between benefits that short-term weather forecasts can provide for improving hydropower generation and the acceptable change in downstream temperatures from the upstream thermal regime.

**Table 4:** Tradeoffs in hydropower generation for a set of constraints of allowable change in temperature

∆*T* (°C) constraint	Hydropower (GWh)	% increase from CLB	∆*T* (°C) constraint	Hydropower (GWh)	% increase from CLB
$\pm \;3$	401.7	− 3.6	$\pm \;6$	472.2	9.3
$\pm \;3.4$	450.8	4.3	No constraint	471.7	9.2
$\pm \;4$	466.3	7.9	BAU	445.1	3.0
$\pm \;5$	471.5	9.1	*CLB*	*459.4*	–

The benefits are compared in terms of percent increase in generation over the benchmark of CLB scenario for the year 2014

As the temperature constraint becomes more stringent, the potential of generating additional hydropower drops. However, the tradeoff does not follow straightforward linear trend. Comparatively lower hydropower was generated for tighter windows of $\pm$ 3.5 °C and lower. As the constraints were relaxed, a sudden increase in benefits is realized with the trend stabilizing on further increasing the allowable temperature change window until $\pm$ 6 °C. When no temperature constraints were imposed, the benefits were comparable to that obtained using constraints of $\pm$ 5 and $\pm$ 6 °C. This signifies an upper limit on energy benefit using flow forecasts, under hydrometeorological conditions of the considered year.

**Fig. 12:**
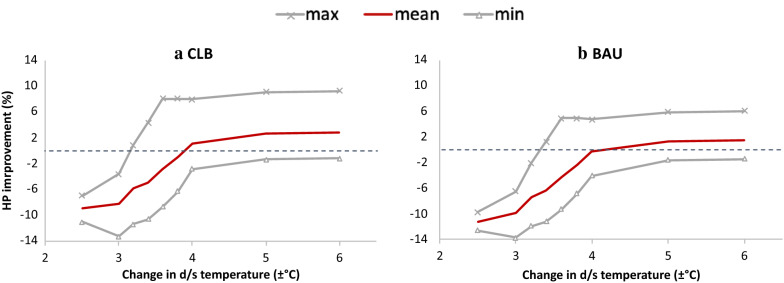
Tradeoff curve for improvement in hydropower generation (HP) over benchmarks of **a** CLB and **b** BAU scenario, with varying temperature constraints. The curve is derived from 5 years of optimization runs performed for Detroit dam involving a series of dry and wet flow regimes

Based on the sensitivity analysis, a tradeoff curve was obtained for Detroit Dam to underscore the obtained improvements in energy generation with varying degrees of allowable temperature change. The multi-objective optimization was performed individually with different constraints of allowable temperature change over 5 years. Years with a blend of dry and wet inflow regimes were selected to arrive at the spread in possible tradeoffs (see Additional file 1: Fig. S2). Figure 12 shows the spread with mean, maximum and minimum of the obtained improvements in hydropower across the selected years. Improvements were calculated against the two benchmark scenarios of CLB and BAU. Hydropower generation (in GWh) for each scenario is shown for each year in detail in Additional file 1: Fig. S3.

The improvement in hydropower benefits generally follows a steep trend in the range of $\pm$ 3 to $\pm$ 4 °C, beyond which the rate of improvement slows down. Comparison against CLB scenario revealed larger improvements as compared to those over BAU scenario. This highlights the value in using real-time inflow forecasts for optimizing reservoir operations against a historical climatology of inflows. The benefits from BAU could also be attributed to other objectives and constraints that operations consider instead of, or in addition to, hydropower and temperature objectives. The loss in hydropower is noticeable with more stringent constraints. The spread in benefits captures the variability in hydrological regime of operations where wetter periods led to larger energy benefits even with stringent constraints. The analysis suggests that beyond a threshold ∆*T* window (around $\pm$ 4 °C for Detroit dam), the amount of additional hydropower benefits is controlled primarily by the skill of weather forecasts and ambient hydrologic conditions. In other words, the dam operators might not benefit much from a wider ∆*T* range as the weather forecasts cannot leverage that high flexibility for improving energy generation. This has far-reaching consequences as even the sensitive ecosystems, where slight alterations to thermal regime can disturb the habitat, can also benefit from weather forecasts while keeping narrow ∆*T* during optimization, depending on the hydrologic conditions.

### Adaptive policy for ecosystem-safe hydropower

**Fig. 13:**
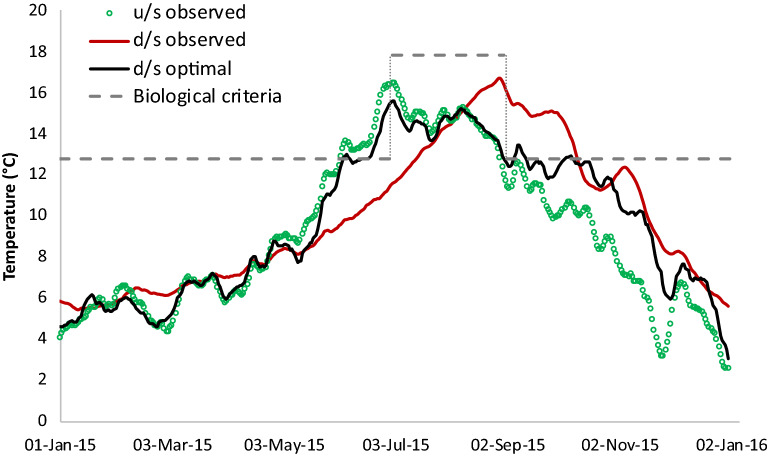
Optimal downstream temperatures during the year 2015 based on the adaptive release policy for Detroit dam. The policy was able to contain downstream temperatures within the required biological criteria to meet spawning and rearing uses, in contrast to the observed scenario exceeding the criteria

Building on from the findings of sensitivity analysis, mimicking natural thermal regime during low water availability is remarkably challenging. In such challenging low flow scenarios, natural temperature is not ensured to produce the best conditions for downstream habitat. We demonstrate an adaptive release policy by moving away from following the upstream temperatures. Over the drought year of 2015 (see Additional file 1: Fig. S2), the multi-objective optimization was performed by embedding biologically based criteria to result in downstream temperatures as shown in Fig. 13.

Under the observed scenario (BAU), temperatures at North Santiam River downstream of Detroit dam exceeded salmon spawning use temperatures from September and into early October. Figure 13 suggests that the adaptive release policy outperformed observed operations and achieved downstream temperatures satisfying the specified biological criteria. Hydropower production over the selected dry year based on adaptive policy amounted to 236.4 GWh, which does not differ significantly in comparison to 235.7 GWh and 240.0 GWh from CLB and BAU scenarios, respectively. This further explains that while it might be challenging to generate additional energy during anomalously dry years, it is indeed possible to meet the biological requirements with proposed adaptive policy.

## Discussion

Our study has found that the objective of hydropower maximization has an aggravating effect on downstream temperatures leading to higher deviation from natural thermal regime upstream. This has implications especially during the peaking power operations when large penstock releases are required while maintaining higher storage levels. During the spawning and rearing periods for certain fish species present in the downstream river reaches, the temperatures have to be maintained within a narrow window which can be challenging to achieve. The multi-objective optimization proposed here provides a framework to incorporate temperature targets while meeting hydropower demands. The tradeoff curves (Fig. 12) facilitate broadening the perspective in comparing different operating policies by informing the dam operator on consequences to ecology and energy generation. Different scenarios can be chosen based on the time of year and optimal conditions for the downstream habitat. The analysis characterizes a reservoir system’s resilience against hydropower operations as well as alterations in river temperature due to climate change impacts.

The functional regression model for modeling the change in thermal regime was found to perform well in capturing the peaks and daily trends over multiple years of data. The simple yet effective regression model allows for its generalized use over any other dam site, with minimal in situ data requirements. The model can be simulated using the global-scale NWP weather forecast fields, while other decision variables of release rates, hydropower generation and elevation are outputs of the reservoir operation model and inflow derived from the flow forecasting model. The performance was comparable to results from other studies using more complex models. A two-layer stratified reservoir model used by Niemeyer et al. (2018) to model absolute river temperatures resulted in RMSE of 1 to 3 °C. Buccola et al. (2016) used a previously calibrated hydrodynamic and water-quality model CE-QUAL-W2 over Detroit dam and reported MAE of 0.34 °C in daily downstream temperatures. Neumann et al. (2003) used a regression model for daily maximum temperature, producing *R*^2^ values of 0.57 to 0.74 over Truckee River. As the aquatic habitat is more sensitive to the changes in temperature instead of the absolute values, simpler regression models present a viable preference for predicting relative changes and integrating with the multi-objective optimization framework. Given that the available temperature models require intensive hydrological and meteorological data and computational effort in model building and calibration, our proposed solution can solve the logistical constraints for data- and resource-constrained settings, especially in the developing nations.

The release decisions ensued from the proposed optimization strategy were in accordance with the downstream flow needed for the specified temperature change constraint. Most of the past studies considered an explicit goal of releasing only environmental flows to imitate the natural flow regime and benefit the native fishes. However, as shown by Chen and Olden (2017), mimicking the natural flow paradigm does not necessarily result in highest benefits. In contrast, our coupled hydropower–temperature based optimization goes beyond the notion of explicitly matching certain flows. It prescribes releases that not only adhere to the best suited environmental flows but is also optimal for hydropower objective. As the water availability continues to shrink, strict objective to meet a pre-specified flow can inherently preclude ability to tailor the downstream conditions on a short-term (e.g., day-to-day) basis. Our results suggest that integrating the ecologically driven objectives (or constraints) within the dam operation module where one has a direct bearing on the other can potentially overcome dam’s detrimental impacts on ecology.

The remote sensing-based results for extracting water surface temperature were encouraging especially for data-constrained regions. The sheer prevalence of planned and under construction hydropower dams in the developing nations (Zarfl et al. 2015) creates a significant opportunity for implementing the concept. Using remotely sensed TIR images for stream temperatures provides an alternative to scarce in situ sensors in such regions for establishing the functional regression models. The technique is not limited to validating regression relations for a particular dam. It can also be incorporated for prior reconnaissance of existing and numerous future dams facing highest degrees of thermal pollution to guide and improve the policy development. It is noteworthy of mention that the approach is limited when mixed water pixels are present in the TIR band. Our results underscored reliable performance for river channels with smaller depth-to-width ratio and where the width covers at least two to three pixels of Landsat ETM+. Similar findings on minimum number of pixels were reported by Handcock et al. (2012) who tested with airborne and satellite TIR images of varying pixel sizes.

In light of the findings from sensitivity analysis, drier years posed challenges in mimicking natural temperature regime. Embedding prescribed biological criteria in the optimization framework with skillful flow forecasts aided in realizing temperatures suitable for relevant downstream aquatic habitat and potentially benefitting energy and ecosystem objectives. USACE recently proposed a temperature control tower for Detroit dam to improve fish passage and temperatures for endangered salmon and steelhead, costing more than USD $350 million (Harrison 2019). Our daily forecast-based optimization demonstrated here can help avoid such expensive measures for other existing and planned dams by minimizing the adverse impacts of dam operations.

## Conclusion

The advancements in flow and reservoir operations modeling have resulted in considerable understanding of the ecological effects of flow regime alteration. This study specifically focused on the thermal impacts of hydropower dams and explored them in the context of operations for improving hydropower generation. Studying the relation between hydropower and changes in thermal regime between dam’s upstream and downstream reaches was facilitated by a simple functional regression model. A multi-objective optimization framework was designed to utilize short-term NWP weather forecasts. The model for water temperature change was used to impose additional constraints of tolerable downstream cooling or warming on multi-objective optimization to maximize hydropower. Remote sensing-based temperature estimation algorithm, valuable for regions with scarce in situ data, was established using thermal infrared band of Landsat ETM+ over multiple dams. The hydropower benefits correlated strongly with the allowable flexibility in temperature constraints. Across the different years with varying climatological conditions, wet years showed maximum hydropower benefits while still satisfying stringent temperature constraints.

It is worth noticing that confidence in the presented tradeoffs in ecosystem-safe hydropower is bounded by the validity of developed hydropower–temperature relationships and flow forecasting model. The performance is affected by how well the temperature changes are modeled as a function of dam operations or if there were any spurious correlations driving the performance (Chen and Olden 2017). Accounting for nonlinear variable relationships in hydro-climatic processes and reservoir’s thermal response is warranted for further improvement. Long-term forecasts (Ahmad and Hossain submitted) can be incorporated to broaden the temporal foresight and operating horizon under climatologically dry/wet years. Finally, for a more holistic ecosystem-safe hydropower optimization, other water quality constituents of concern such as dissolved oxygen levels, total dissolved solids, and bubbling of carbon dioxide or methane from reservoirs also need consideration.

While the cost to environment can never be completely eliminated, we have demonstrated a practicable solution to navigate the tradeoffs in hydropower energy and thermal stability needs of humans and ecosystem. The challenge is now to realize this as an operating standard for existing and future dams and thus foster the goal of clean energy without sacrificing the societal and ecosystem benefits.

## Supplementary information

**Additional file 1.** Additional figures and tables.

## Data Availability

The datasets used and/or analyzed during the current study are available from the corresponding author on reasonable request.
