# Supplementary material for: Realizing ecosystem-safe hydropower from dams
Source: Renew Wind Water Sol. 2020 Jun 1;7(1):2. doi: 10.1186/s40807-020-00060-9 (PMC7325499; doi:10.1186/s40807-020-00060-9)
Supplement: Supplementary file 1 — Additional file 1. Additional figures and tables. [file 40807_2020_60_MOESM1_ESM.docx]

**Additional file 1**

**Selected dams for remote sensing-based temperature estimation**

**Table S1.** Summary of the selected dams, their approximate downstream river channel widths, Landsat-7 ETM+ scene path and row numbers, and USGS upstream and downstream stations for temperature measurements.

| **S. No.** | **Dam name** | **Normal Reservoir depth (m)** | **Down-stream river width (m; approx.)** | **Landsat 7 scene #** | | **USGS station ID(s)** | | **Analysis period** | |
| --- | --- | --- | --- | --- | --- | --- | --- | --- | --- |
|  |  |  |  | **Path** | **Row** | **u/s** | **d/s** | **u/s** | **d/s** |
| 1. | Fort Peck, MT | 67 | 250 | 35 | 27 | 06115200 | 06177000 | 2010-18 | 2002-04 |
| 2. | Palisades, ID | 75 | 100 | 38 | 30 | 09205000 | 13032500 | 2015-19 | 2006-15 |
| 3. | Glen Canyon, AZ | 170 | 150 | 37 | 34 | 09379500 | 09380000 | 2009-16 | 2009-16 |
| 4. | Dworshak, ID | 192 | 75 | 42 | 28 | 13340600 | 13341000 | 2009-18 | 2009-18 |
| 5. | Detroit, OR | 130 | 40 | 45 | 29 | 14179000, 14178000,  14180300 | 14181500 | 2010-19 | 2010-19 |
| 6. | Lower Monumental*, WA | 165 | 300 | 43 | 28 | 13352595 | 13352600 | 2010-19 | 2010-19 |
| 7. | McNary*, WA | 23 | 800 | 44 | 28 | 14019220 | 14019240 | 2010-19 | 2010-19 |
| 8. | Keystone, OK | 22 | 250 | 27 | 35 | 07176950 | 07164500 | 2004-05 | 2009-19 |
| 9. | Green Peter, OR | 100 | 35 | 45 | 29 | 14185900 | 14186200 | 2010-19 | 2010-19 |
| 10. | Holter, MT | 15 | 115 | 39 | 27 | 06054500 | 06066500 | 2010-18 | 2010-18 |

* run-of-river dam

**Hydrological characteristics and performance evaluation over selected years**

**Figure S1.** Flow climatology for Detroit dam, derived using 50 years of reservoir inflow data (1961-2010) (Source: USACE)

**Figure S2.** (a) Daily inflow into Detroit reservoir over selected years of analysis, (b) Corresponding annual inflow rates over each year, dashed line shows climatological average flow rate.

| **Dry** |  |  |  |
| --- | --- | --- | --- |
| **Wet** |  |  | *CLB*: Climatological baseline  *BAU*: Business-As-Usual  *NC*: No temperature constraint |

**Figure S3.** Hydropower generation from different constraints on allowable temperature difference, compared with those from scenarios of no temperature constraint (NC), CLB and BAU, over wet and dry years.
